# Visual Detection of Ascorbic Acid via Smartphone with Portable Probe Based on Assembled FePO_4_@GO Peroxidase-like Nanozyme

**DOI:** 10.3390/molecules29215097

**Published:** 2024-10-29

**Authors:** Zifang Zhang, Junhua Bai, Yan Gong, Lifang Zhang

**Affiliations:** 1 School of Chemistry and Materials Science, Shanxi Normal University, Taiyuan 030031, China; 2 School of Life Science, Shanxi Normal University, Taiyuan 030031, China; 3 Collaborative Innovation Center for Shanxi Advanced Permanent Magnetic Materials and Technology, Taiyuan 030031, China

**Keywords:** FePO_4_@GO nanozymes, peroxidase-like nanozymes, ascorbic acid, visual detection

## Abstract

Ascorbic acid (AA) plays an important role in many physiological and biochemical processes. Adequate quantification of AA is required for controlling the intake of AA for health management. In this study, a portable probe was fabricated based on the assembled FePO_4_@GO nanozyme by homogeneous precipitation. FePO_4_@GO exhibits excellent peroxidase (POD) activity. As an efficient free radical scavenger, ascorbic acid scavenges ^1^O_2_ free radicals produced by hydrogen peroxide catalyzed by peroxidase enzymes and then inhibits the color reaction of 3,3′,5,5′-tetramethylbenzidine (TMB). A good linear relationship between the color of the reaction system and the concentration of AA in the range of 2.5–75 μM was presented, and the detection limit is 1.25 μM. Furthermore, a visual colorimetric detection platform was constructed using a smartphone. The AA content is accurately reflected by intelligent recognition of color changes in the reaction system. It provides an alternative approach for portable and visual rapid detection of Vitamin C (VC) in medicine. Meanwhile, it also provides a reference for the assembly of an intelligent detection platform based on nanozymes.

## 1. Introduction

Enzymes, as a powerful class of catalysts crucial to life processes, are primarily composed of proteins [1,2]. They play a dominant role in regulating biological activities and are also essential catalysts in chemical and industrial domains. Currently, enzymes find extensive application in various sectors, including agriculture, medicine, industry, environmental protection, and life science research [2]. Along with the rapid advancement of science and technology, enzyme engineering is evolving continuously, and its influence extends across multiple fields [3].

Although enzymes have seen widespread use, they still exhibit significant shortcomings, including poor stability, difficulties in preservation, high sensitivity to environmental conditions, and challenges in recycling. These limitations constrain their extensive application in areas like food processing and biochemical detection [4,5]. In recent years, the quest for artificial enzymes has ignited considerable interest among researchers. The discovery of inherent peroxidase activity in ferromagnetic nanoparticles dates back to 2007 [6]. Since then, the exploration of artificial enzyme mimics has garnered significant attention among researchers, particularly given the rapid advancements in nanotechnology and nanomaterials [3,7,8]. Artificial mimicry of enzymes at the nanoscale, often referred to as nanozymes, has become a prominent area of study and application [2]. Nanozymes represent a category of materials with enzymatic catalytic activity, characterized by their sizes falling within the range of 1 to 100 nm. When compared to natural enzymes, nanozymes offer several distinct advantages, including exceptional stability, tunable and notably high catalytic activity [7,8], lower sensitivity to variations in temperature and pH, recyclability, and a broader spectrum of applications, such as biosensing, environmental remediation, and disease diagnosis [9,10].

As a nanozyme, FePO_4_ exhibits immense potential for rapid detection and analysis [11,12,13]. Under acidic conditions, FePO_4_ displays robust peroxidase activity, while under neutral conditions, it demonstrates significant catalase-like activity [14]. In the absence of hydrogen peroxide (H_2_O_2_), FePO_4_ exhibits excellent biocompatibility, but with the introduction of H_2_O_2_, it manifests potent catalytic activity with high sensitivity and rapid reaction kinetics [15,16].

Using its advantages in nanoelectronics, composite materials, and biosensing, graphene oxide (GO) has garnered the attention of numerous scholars in recent years, resulting in significant progress [17,18,19]. GO offers features like a large specific surface area, cost-effective preparation, and low toxicity [20,21]. Its distinctive two-dimensional layered structure and excellent biocompatibility render it an outstanding nanomaterial carrier and a precursor for inorganic nanomaterials [12,22]. The composite materials, such as GO combined with TiO_2_ or Ag, find applications in photocatalysis, exhibiting exceptional catalytic activity facilitated by GO’s role as a sensitizer, which enhances catalytic absorption rates [23,24,25]. Numerous studies have confirmed GO’s peroxidase activity, which has been widely explored [17,22,26].

Ascorbic acid (AA) plays a crucial role in the body’s metabolic processes, facilitating cell growth, calcium absorption, and the development of normal tissue [27,28]. It is abundantly found in fruits, and its concentration is regarded as an important indicator of fruit freshness and nutritional quality [29]. Up to now, AA has primarily been detected by using methods, such as liquid chromatography, fluorescence spectroscopy [29,30], and electrochemical techniques [31]. However, these detection methods are often complex to operate and the instruments are not easily portable and suitable for on-site detection. Smartphones have the advantages in universality, convenience, and high-resolution imaging, and many digital and intelligent POCT systems have been developed based on their advantages [32] in terms of convenience, portability, visual observation, and on-site detection that make them an optimal strategy for detection. It is anticipated that this technology will find extensive applications in biosensing, food safety assurance, environmental protection, and industrial processes.

**Scheme 1:**
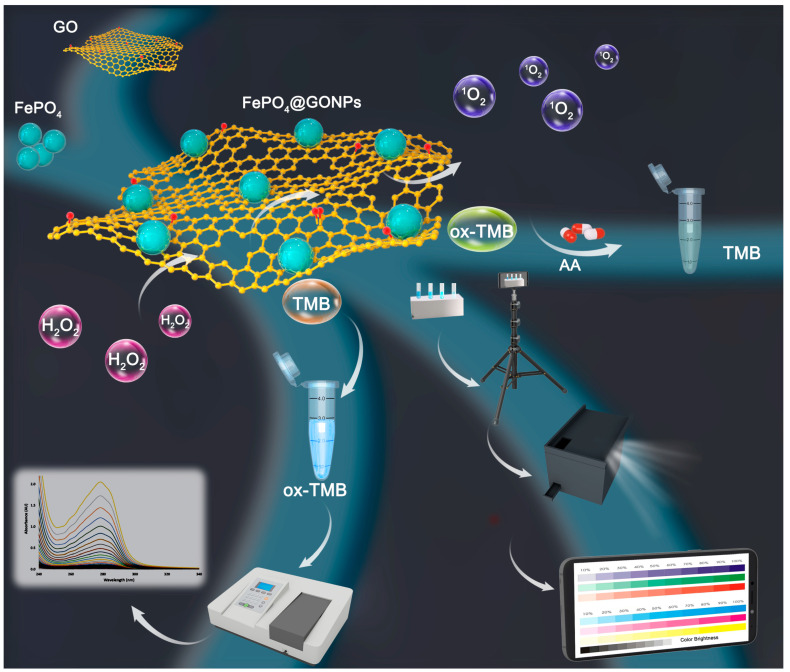
Based on the peroxidase-like activity of FePO_4_@GO NPs, the portable biosensor designed by UV-Vis and smartphone colorimetric software (Color Collect Pro 2.5.36) was used to accurately detect AA.

In this study, we employed a straightforward homogeneous precipitation process to fabricate an FePO_4_@GO nanoparticles (NPs)NPs composite material mimicking peroxidase. Serving as an excellent sensitizer, GO offers optimal adsorption and reaction sites due to its high specific surface area, thereby enhancing FePO_4_’s catalytic activity. We investigated FePO_4_@GO NPs’s catalytic efficiency in the presence of H_2_O_2_ for the colorimetric substrate TMB, revealing its strong affinity for TMB and peroxide-like catalytic activity. Using the remarkable peroxidase properties of FePO_4_@GO NPs, we employed it in an antioxidant study involving AA. In this study, oxidized TMB (oxTMB) was effectively reduced back to TMB through a double-electron transfer mechanism, resulting in the fading of the blue color associated with oxTMB. Building upon this principle, we designed a signal-sensing platform that offers portable and visually accessible detection capabilities, utilizing the high-quality camera functions of modern smartphones (Scheme 1).

## 2. Results

### 2.1. Characterizations of FePO_4_@GO NPs

**Figure 1:**
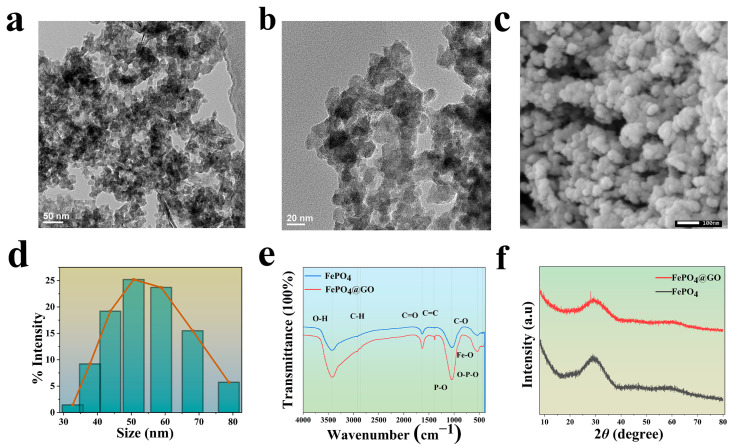
(**a**). TEM images of FePO_4_@GO NPs. (**b**). HR-TEM images of FePO_4_@GO NPs. (**c**). SEM images of FePO_4_@GO NPs. (**d**). The size distribution of the FePO_4_@GO NPs. (**e**). FT-IR spectrum of the FePO_4_@GO NPs and FePO_4_. (**f**). XRD patterns of FePO_4_@GO NPs and FePO_4_.

As depicted in Figure 1a,b, the obtained TEM and HR-TEM images of FePO_4_@GO NPs exhibited a layered structure. Doping with nanospheres of FePO_4_ effectively prevented the aggregation of GO, creating space for the peroxidase reaction system [33]. Figure 1c reveals SEM images of FePO_4_@GO NPs with a spherical structure [34], which closely interacts with the embedded GO [11,15,34]. The particle size of FePO_4_@GO NPs, as evident in Figure 1a–c, measures approximately 30–50 nm, with an average diameter of 50.9 nm according to the test results (Figure 1d). FT-IR spectroscopy was employed to analyze the functional groups of the composite and FePO_4_. Figure 1e reveals various peaks, including a stretching vibration peak at 3423 cm^−1^ attributed to hydroxyl, two stretching vibration peaks at 2926 cm^−1^ and 2856 cm^−1^ for the C-H bonds in the alkyl group, and a vibration peak at 1634 cm^−1^ associated with water. The peak at 1456 cm^−1^ (C=C) indicates the successful combination of GO and FePO_4_ [35,36]. A strong peak at 1046 cm^−1^ may correspond to the antisymmetric stretching vibration of P-O in the phosphate group or the stretching vibration of the C-O bond. Similarly, the antisymmetric bending vibration peak of O-P-O and the correlation peak of Fe-O bonds appear at 539 cm^−1^ and 430 cm^−1^, respectively, confirming the successful synthesis of FePO_4_ in the reaction system [35,37,38]. The successful preparation of FePO_4_@GO NPs and FePO_4_ was confirmed through XRD measurements. As illustrated in Figure 1f, no distinct diffraction peaks attributed to FePO_4_@GO NPs and FePO_4_ are observed, suggesting that the composite exists in a non-crystalline state. This characteristic may be closely linked to the low temperature maintained during the reaction process [39]. The crystalline state is known to have a certain correlation with peroxidase activity. Typically, FePO_4_@GO NPs exhibit low activity when in a crystalline state. Figure analysis indicates that the diffraction peaks of GO are not prominent and this can be attributed to two possible factors. First, the relatively low content of GO in the composite may contribute to this effect. Second, a significant quantity of FePO_4_ may be adsorbed on the surface of GO, which could influence the XRD diffraction peaks [39].

### 2.2. Peroxidase-like Activity of FePO_4_@GO NPs

**Figure 2:**
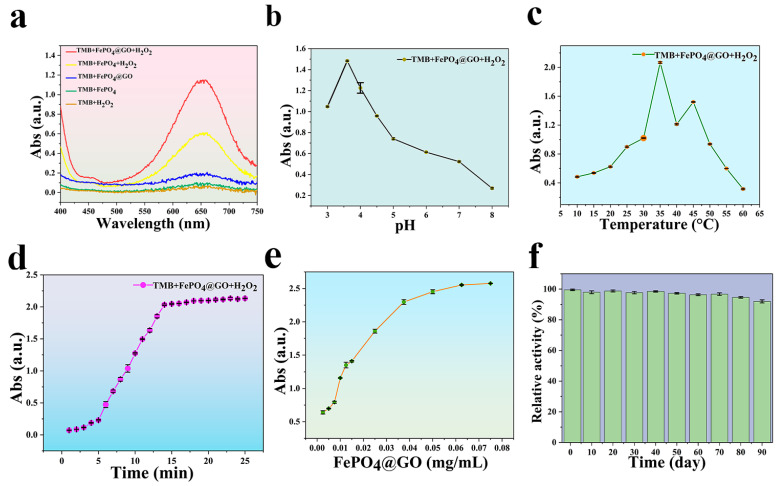
(**a**). The UV-Vis absorption spectra of different colorimetric reaction systems. (**b**). The effect of buffer pH on the POD-like activity of FePO_4_@GO NPs. (**c**). Temperature-dependence of FePO_4_@GO NPs. (**d**). The effect of time on POD-like activity of FePO_4_@GO NPs. (**e**). The effect of the concentration of FePO_4_@GO NPs on POD-like activity. (**f**). The catalytic stability evaluation of FePO_4_@GO NPs for different times.

In the study of peroxidase-like activity, FePO_4_@GO NPs catalyzed the oxidation of the typical peroxidase substrate TMB in the presence of H_2_O_2_ and the presence of an obvious absorption peak at 652 nm in the UV-Vis spectrum (Figure 2a) indicated an oxidation reaction when FePO_4_@GO NPs catalyzed TMB in the presence of H_2_O_2_. When any one of FePO_4_@GO NPs, TMB, or H_2_O_2_ was absent, no significant color change or absorption peak was observed. Additionally, FePO_4_ catalyzed the TMB in the presence of H_2_O_2_. This experiment revealed that the catalytic activity of FePO_4_@GO NPs was notably superior to that of FePO_4_. These findings provide evidence that FePO_4_ and GO jointly enhance the peroxidase activity of FePO_4_@GO NPs through a synergistic reaction.

Based on the experimental investigation, the peroxidase-like activity of FePO_4_@GO NPs in the entire catalytic system was found to be influenced by various factors, including temperature, pH value, enzyme concentration, and catalytic time. Therefore, each of these factors was optimized individually in the FePO_4_@GO NPs-based catalytic system. Figure 2b illustrates that FePO_4_@GO NPs displayed the best peroxidase catalytic activity when the catalytic system’s pH was maintained at 3.6 using a buffer solution. As depicted in Figure 2c, the optimal catalytic temperature for FePO_4_@GO NPs was 35 °C in a pH 3.6 environment. Under these conditions, a water bath was employed to stabilize the reaction solution at 35 °C, and the FePO_4_@GO NPs’s peroxidase catalytic time was recorded. Figure 2d reveals that under the optimal reaction environment, the absorbance change stabilized after 15 min of catalysis by FePO_4_@GO NPs. The absorbance change pattern aligns with the characteristics of enzyme kinetic reactions, with the best catalytic time determined to be 15 min. Additionally, we investigated the impact of FePO_4_@GO NPs concentration on peroxidase activity (Figure 2e). The results reveal that as the concentration of FePO_4_@GO NPs increased, the activity of FePO_4_@GO NPs gradually improved, reaching its peak and stabilizing at a concentration of 0.0625 mg/mL.

To assess the stability of FePO_4_@GO NPs, we conducted tests on samples stored at 4 °C for periods ranging from 1 to 90 days. The results demonstrate that FePO_4_@GO NPs exhibited excellent stability, with peroxidase activity showing minimal variation compared to freshly prepared samples (Figure 2f).

### 2.3. Steady-State Kinetics of FePO_4_@GO NPs

**Figure 3:**
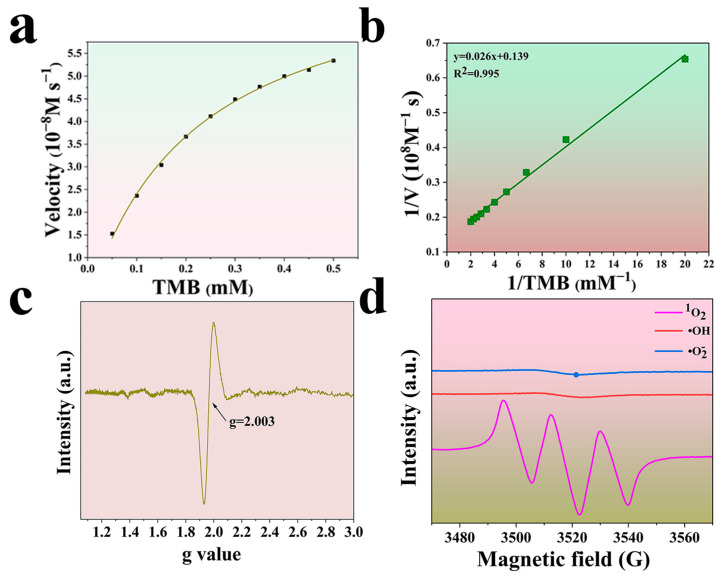
(**a**). Michaelis–Menten curves of FePO_4_@GO NPs. (**b**). Lineweaver–Burk curve of FePO_4_@GO NPs for TMB. (**c**). EPR spectrum of FePO_4_@GO NPs samples. (**d**). EPR spectrum of FePO_4_@GO NPs + H_2_O_2_ + TEMP/DMPO.

To investigate the peroxidase kinetics of FePO_4_@GO NPs, TMB was employed as the reaction substrate and the catalytic reaction was conducted under optimal reaction conditions. As depicted in Figure 3a, when employing TMB as the substrate for FePO_4_@GO NPs, the curve model could be successfully fitted using the Michaelis–Menten equation [32,40,41]. Utilizing the Lineweaver–Burk plotting method, as presented in Figure 3b, allowed for a more accurate determination of the *K*_m_ and *V*_max_ values, which were found to be 0.2202 mM and 7.71219 × 10^−8^ Ms^−1^, respectively. The linear and nonlinear fitting indices were 0.995 and 0.998, respectively, reflecting extremely high accuracy. When compared to previous studies and typical natural horseradish peroxidases (HRP) [41] (Table S1), FePO_4_@GO NPs exhibited a lower *K*_m_ value and a similar *V*_max_ value, indicating robust peroxidase activity.

### 2.4. Catalytic Mechanism

Herein, D-Histidine, BQ, IPA, and EDTA were employed as scavengers to analyze ROS [40,42]. As illustrated in Figure S1, in contrast to reactions without ROS scavengers or with the addition of ROS scavengers, the activity of FePO_4_@GO NPs decreased upon adding D-Histidine or EDTA. The addition of EDTA led to a significant reduction in activity, suggesting that oxygen vacancies (OVs) OVs played a major role in the catalytic process when FePO_4_@GO NPs were used as a peroxidase. Furthermore, ^1^O_2_ also played a role in promoting the catalytic process. When D-Histidine and EDTA were simultaneously added to the reaction system containing FePO_4_@GO NPs, TMB, H_2_O_2_, and buffer, the activity of FePO_4_@GO NPs nearly disappeared completely.

Based on these findings, the generation of ^1^O_2_ and OVs was confirmed through electron EPR spectroscopy. The presence of OVs in FePO_4_@GO NPs was indicated by the appearance of their characteristic EPR signal peak at g = 2.003 (Figure 3c). OVs in FePO_4_@GO NPs can induce active sites of the nanozyme and enhance the peroxidase activity of FePO_4_@GO NPs.

DMPO was used as the capture agent for ·OH (0.3 M, in ethanol), and ·O_2_^−^ (0.3 M in methanol), while TEMP (0.25 M, in ethanol) was employed as the capture agent for ^1^O_2_. As demonstrated in Figure 3d, under the conditions where FePO_4_@GO NPs, H_2_O_2_, and TEMP co-existed, a strong characteristic peak with a 1:1:1 ratio appeared, representing a typical signal peak of ^1^O_2_ [43]. This further confirmed the generation of ^1^O_2_ catalyzed by H_2_O_2_.

### 2.5. Detection of AA by FePO_4_@GO NPs

**Figure 4:**
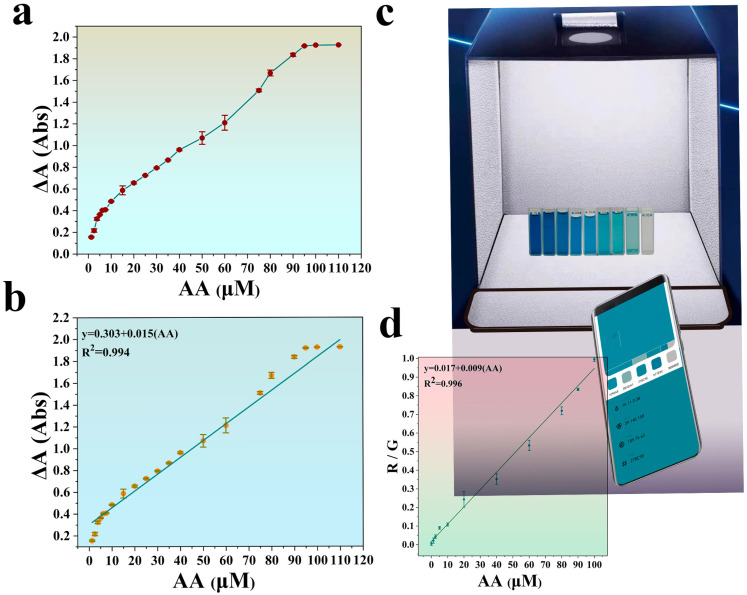
(**a**). UV-Vis absorbance change by different concentrations. AA was added to the reaction system TMB + FePO_4_@GO NPs + H_2_O_2_ catalyzed for 15 min. (**b**). The typical linear calibrations. (**c**). Smartphone photography and Red, Green, Blue (RGB) RGB information collection. (**d**). The relative color information (R/G) versus concentration of AA.

FePO_4_@GO NPs with POD activity could oxidize TMB to ox-TMB, producing an absorption peak at 652 nm, resulting in a blue color change in the solution. AA possessed strong reducibility and acted as a typical antioxidant. After adding AA, the absorption peak at 652 nm decreased, causing the solution to fade. The experimental results indicate that the absorption peak decreased progressively with the addition of different concentrations of AA. The absorbance change (ΔA) is calculated by A (652 nm, absence)-A (652 nm, AA). As shown in Figure 4a, the linear range could be controlled within 2.5–75 μM (Figure 4b). Therefore, the FePO_4_@GO NPs-mediated ox-TMB-based detection system could be employed as an amplification method for detecting AA. Additionally, interference tests were conducted using various interfering substances, revealing no significant changes in the absorption peak (Figure S2), indicating that FePO_4_@GO NPs exhibited good selectivity and sensitivity to AA.

To assess the reliability of FePO_4_@GO NPs in detecting AA, repeated experiments were conducted in different environments. Eight samples were selected for each group of experiments, with a total of five group tests. The results show minimal differences between the experimental results within the same batch and among different batches, with the RSD value staying within 4%. Furthermore, we performed a recycling experiment on FePO_4_@GO NPs after the detection of AA. The samples were washed, filtered, dried, and subsequently once more detected using FePO_4_@GO NPs. The results indicate (Figure S3) that the catalytic activity of FePO_4_@GO NPs remained almost unchanged. In addition, we compared the XRD measurements of FePO_4_@GO NPs before and after testing (Figure S4). The XRD diffraction peaks exhibited no significant alterations before and after the experiment, suggesting that the crystal structure of FePO_4_@GO NPs was not changed. This indicated that FePO_4_@GO NPs had strong reliability in the detection of AA.

### 2.6. Portable Detection of Real Samples Based on Smartphones

To validate the reliability of FePO_4_@GO NPs in detecting the concentration of AA in real samples, VC was chosen as the real sample, and the test results show that the content of AA was 93 mg/g. This value closely matched the number displayed (100 mg/g) on the product information table label. The relative standard deviation (RSD) obtained by comparing the concentration of AA detected by UV-Vis spectrophotometry and the specified value in the specification was 3.43% (n = 5), demonstrating the high reliability of the detection method.

To make the detection of AA more convenient, a method for detecting AA based on FePO_4_@GO NPs using smartphones was developed. As shown in Figure 4c, the color of the reaction system changed with variations in the concentration of AA. With the increasing concentration of AA, the blue solution gradually lightened. Since each color has a fixed RGB value, it can be easily read using the camera function of smartphones (honor magic5, SmartTrust New Information Technology Co., Ltd., Shenzhen, China). However, different colors can be influenced by light during the photography process, potentially causing errors. To mitigate this, we established a relatively enclosed environment using a simple, portable studio. In this setup, we employed a mobile power supply to generate a consistent light source with fixed brightness. When taking photos, the smartphone’s camera function was utilized to capture images, and professional color collection app software (Color Collect Pro 2.5.36) was employed to digitize the RGB values for obtaining the corresponding values. R/G value and AA concentration were chosen for linear fitting (Figure 4d), resulting in a regression equation of y = 0.01746 + 0.0093 [AA] and R^2^ = 0.99624. Similarly, when comparing the obtained results with standard values, an RSD value of 4.58% was achieved, affirming the applicability and reliability of the portable detection platform based on FePO_4_@GO NPs.

### 2.7. Advantages of Probe

FePO_4_@GO NPs exhibit excellent peroxidase activity, and the portable probe constructed by using FePO_4_@GO exhibits a broader detection range and a lower detection limit compared with recently reported detection methodologies (Table S2). In addition, the portable detection of AA based on smartphones has high reliability, and this methodology offers several significant analytical advantages, including long-term stability, good selectivity, rapid detection and suitability for on-site detection.

## 3. Materials and Methods

### 3.1. Materials

Graphite powder (325 mesh, 99.99% purity) came from Alfa Aesar (China) Chemical reagent Co., Ltd. (Shanghai, China). Ascorbic acid (AA) and dimethyl sulfoxide (DMSO) were obtained from Tianjin Berens Biotechnology Co., Ltd. (Tianjin, China). Potassium persulfate (K_2_S_2_O_8_), iron (III) nitrate Fe(NO_3_)_3_, sodium hydroxide (NaOH), phosphorus pentoxide (P_2_O_5_), nitric acid (HNO_3_), and concentrated sulfuric acid (H_2_SO_4_, 95%) were purchased from Merck. Ammonium dihydrogen phosphate (NH_4_H_2_PO_4_, 99%), 3,3′,5,5′-tetramethylbenzidine (TMB), horseradish peroxidase (HRP), 2,2,6,6-tetramethylpiperidine (TEMP), ethanol absolute, 1,4-Benzoquinone (BQ), isopropanol (IPA), horseradish peroxidases (HRP, >300 units/mg), d-Histidine, sodium acetate (NaAc, 99%), ethylenediaminetetraacetic acid (EDTA, ≥99.5%), fructose (99%), and 5,5-dimethyl-1-pyrroline N-oxide (DMPO, 97%) came from Aladdin Biochemical Technology Co., Ltd. (Shanghai, China). Methanol, hydrogen peroxide (H_2_O_2_, 30%), and potassium chloride (99%) came from Luoyang Chemical Reagent Factory. L-Lysine (98%), galactose (99%), cysteine (98%), glucose (99%), maltose (98%), leucine, phenylalanine (99%), threonine (99.9%), and methyl orange came from Beijing Bailingwei Technology Co., Ltd. (Beijing, China). Acetic acid (HAc, ≥99.5%) was obtained from Sinopharm Chemical. Vitamin C chewable tablets (VC) came from Guangdong Changxing Biotechnology Co., Ltd. (Chaozhou, China). Ultrapure water (≥18.2 MΩ·cm) from an Elix10/Milli-Q system (Millipore, St. Louis, MO, USA) was applied for solution preparation.

### 3.2. Synthesis of FePO_4_@GO NPs

GO was synthesized by the standard Hummer method [12,33,44]. Briefly, 10 g of graphite powder, 10 g of potassium persulfate (K_2_S_2_O_8_), and 10 g of phosphorus pentoxide (P_2_O_5_) were added to a triangular flask. Then, 50 mL of concentrated sulfuric acid (H_2_SO_4_) was slowly added into the above triangular flask. After stirring for 10 min, the dispersion was heated to 80 °C for 6 h, then slowly cooled to room temperature. Subsequently, the mixture was diluted with ultrapure water, followed by filtration and washing. The obtained solid was then dried overnight in vacuum under 70 °C.

FePO_4_@GO NPs were synthesized by the homogeneous precipitation process. As-prepared GO was put in 100 mL of ultrapure water in an ultrasonic bath for 1 h; the addition amount of GO was based on the total mass of NH_4_H_2_PO_4_ and Fe(NO_3_)_3_ in the system, which was taken as the proportions of 25%. Then, 25 mL of NH_4_H_2_PO_4_ solution (90 mg/mL) and 25 mL of Fe(NO_3_)_3_ (323 mg/mL) solution were added into the above GO solution. The pH of solution was adjusted to 1.5 using 0.1 M nitric acid aqueous solution. After continuously stirring at 600 rpm for 1 h at 70 °C, the mixture was allowed to stand for 12 h. The mixture was then centrifuged, and the resulting precipitate was washed three times with ultrapure water and ethanol. Finally, the prepared composite material was dried overnight at 70 °C in a vacuum drying oven.

### 3.3. Characterization of FePO_4_@GO NPs

The morphologies of samples were observed using a transmission electron microscope (TEM, Tecnai G2 F30, Eindhoven, The Netherlands), high-resolution transmission electron microscope (HRTEM, iTecnai F30, Eindhoven, The Netherlands), and scanning electron microscope (SEM, JSM-IT800, Tokyo, Japan). Surface functional groups and chemical bonds of samples were recorded by Fourier transform infrared spectroscopy (FT-IR, Varian 660, New York, NY, USA). ROS were captured using electron paramagnetic resonance (EPR, Bruker A300, Karlsruhe, Germany). X-ray diffraction (XRD) results were obtained by an X-ray diffractometer (Ultima IV-185, Tokyo, Japan) using Cu Kα radiation (40 kV, 25 mA); the scanning range was from 10° to 80°, and the scanning rate was 5° min^−1^. Dynamic light scattering analyzer (DLS, Zetasizer NanoZS, Malvern, UK) was used to detect the particle size distribution of nanoparticles. The change in absorbance of TMB solution during the peroxidase reaction was recorded using a UV-Vis spectrophotometer (UV-3200PC, Shanghai, China) at 652 nm. pH value was measured at room temperature with a pH meter.

### 3.4. Simulation and Comparison of Peroxidase Activity of FePO_4_@GO NPs

TMB served as the chromogenic substrate, and HAc–NaAc buffers with varying pH levels were employed as the reaction medium. The classic chromogenic reaction involving H_2_O_2_ and TMB was utilized to assess the peroxidase activity change. The optimized reaction system consisted of 1 mg/mL FePO_4_/FePO_4_@GO NPs suspension, 200 µL of 0.1 M H_2_O_2_ solution, and 200 µL of 10 mM TMB chromogenic solution mixed in 3.4 mL of HAc–NaAc buffer with different pH values. After a 10 min incubation, the absorbance of the mixture at 652 nm was measured using a UV-Vis spectrophotometer. The impact of temperature and pH was investigated by controlling these variables separately. Additionally, FePO_4_@GO NPs were synthesized and subsequently incubated in a refrigerator and at room temperature for six months to assess the stability of their peroxidase activity.

The initial reaction velocity (*v*) and substrate concentration (*S*) were experimentally calculated using the Michaelis–Menten kinetics under the quantitative condition of FePO_4_@GO NPs. The kinetic parametric equation is as follows:

(1) $$v=\frac{v_{max}\left[S\right]}{K_{m}+\left[S\right]}$$

The maximum reaction rate (*V*_max_) and the reaction constant (*K*_m_) of Michaelis–Menten kinetics were determined using Lineweaver–Burk double reciprocal graphs based on first-order, second-order, and zero-order reaction processes. The equation used for this analysis is as follows:

(2) $$\frac{1}{v}=\frac{K_{m}}{V_{max}\left[S\right]}+\frac{1}{v_{max}}\;$$

where *K*_m_ represents the substrate’s affinity for peroxidase, which corresponds to half the reaction rate at maximum substrate concentration, (*S*) denotes the substrate concentration (TMB or H_2_O_2_), *V*_max_ signifies the maximum reaction rate achieved at saturated substrate concentration, and *V* represents the initial reaction rate.

### 3.5. Catalytic Mechanism of FePO_4_@GO NPs as Peroxidase-like Activity

To investigate FePO_4_@GO NPs’s catalytic mechanism as a peroxidase mimic, various free radical scavengers were employed to confirm the production of reactive oxygen species (ROS) within FePO_4_@GO NPs. D-Histidine, 1,4-benzoquinone (BQ), isopropanol (IPA), and ethylenediaminetetraacetic acid (EDTA) were used as scavengers for singlet oxygen (^1^O_2_), superoxide anion radical (O_2_^·−^), hydroxyl radical (·OH), and oxygen vacancies (OVs), respectively. Since TMB, o-Phenylenediamine (OPD), and 2, 2′-azino-bis (3-ethylbenzothiazoline 6-sulfonic acid) (ABTS) react with BQ during the reaction, we used methyl orange (MO) instead of TMB as the colorimetric substrate for comparison [45,46]. The change in absorbance at 652 nm upon scavenger addition was recorded using a UV-Vis spectrophotometer. Furthermore, to further ascertain reactive oxygen species (ROS) production by FePO_4_@GO NPs as a peroxidase mimic during the catalytic reaction, 2,2,6,6-tetramethylpiperidine (TEMP) was employed as the capture agent for ^1^O_2_, while 5,5-dimethyl-1-pyrroline N-oxide (DMPO) was used as the capture agent for ·OH and O_2_^·−^. The generation of ROS was detected via electron paramagnetic resonance (EPR) spectroscopy.

### 3.6. Detection of AA

Different concentrations of AA were added to the HAc–-NaAc buffer (pH = 3.6), with fixed concentrations of FePO_4_@GO NPs, H_2_O_2_, and TMB. The change in AA concentration within the range of 0–0.5 mM was observed. The change in absorbance of the solution is the difference between the absorbance values before and after the addition of a known quantity of AA. The absorbance change (ΔA) is calculated by A (652 nm, absence)-A (652 nm, AA) and measured at 652 nm following a 15 min incubation period.

## 4. Conclusions

In conclusion, we successfully developed a novel nanomaterial comprised of FePO_4_ supported by GO, exhibiting outstanding POD-like activity. This nanomaterial has the unique capability to generate ^1^O_2_ through the catalysis of H_2_O_2_ by its inherent OVs. In stability kinetic tests, it displayed a low *K*_m_ value and a *V*_max_ value similar to that of natural enzymes, confirming its high and consistent POD activity. We also introduced a novel detection method for AA based on the reaction between FePO_4_@GO NPs and the ROS generated. Additionally, we created a portable and rapid mobile phone sensing platform with the assistance of smartphones. Our results demonstrate that the colorimetric sensing detection method, relying on FePO_4_@GO NPs and detected by smartphones, offers numerous advantages, including result stability, simplicity of operation, cost-effectiveness, and convenient portability. This platform represents a practical and user-friendly on-site intelligent detection solution and has great potential for the development in the fields of food safety and the environment. Furthermore, FePO_4_@GO NPs recycling still depends on manual operations and requires tedious experiments. In order to enable simple, rapid recycling, we will develop magnetic composites based on high enzyme activity for better reuse in our future work.

## Data Availability

The original contributions presented in this study are included in the article and Supplementary Materials. Further inquiries can be directed to the corresponding author.

## Supplementary Materials

The following supporting information can be downloaded at: https://www.mdpi.com/article/10.3390/molecules29215097/s1, Figure S1: The relative POD-like activity of FePO_4_@GO NPs catalytic system with ROS scavengers (IPA, BQ, D-Histidine and EDTA, 5 mM; FePO_4_@GO NPs: 0.10 mg/mL, TMB:1.0 mM, H_2_O_2_: 0.5 mM, pH 3.6. Inset: color changes of TMB in four samples); Figure S2: The selectivity of FePO_4_@GO NPs for AA detection. (1 mM AA, 10 mM interfere materials); Figure S3: The UV-vis absorption spectra of recycling experiment on FePO_4_@GO NPs; Figure S4: The XRD patterns of recycling experiment on FePO_4_@GO NPs; Table S1: Comparison of the Michaelis-Menten constant (K_m_) and the maximum velocity (V_max_) of FePO_4_@GO NPs with other nanomaterial; Table S2: Comparison of AA detection with different methods. Refs. [47,48,49,50,51,52] are cited in Supplementary Materials.
