# Supplementary material for: Visual Detection of Ascorbic Acid via Smartphone with Portable Probe Based on Assembled FePO4@GO Peroxidase-like Nanozyme
Source: Molecules. 2024 Oct 29;29(21):5097. doi: 10.3390/molecules29215097 (PMC11547973; doi:10.3390/molecules29215097)
Supplement: Supplementary file 1 [file molecules-29-05097-s001.zip › molecules-3157886-supplementary.pdf]

Figure S1.

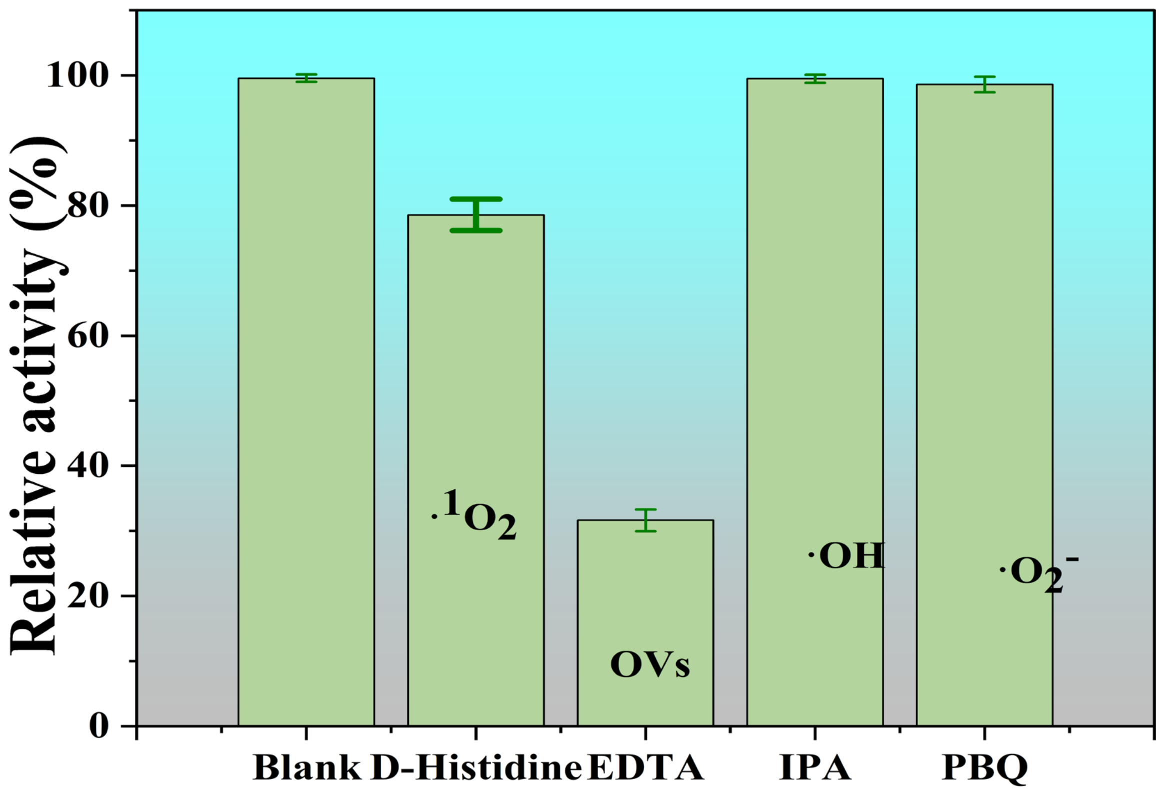

Figure S1. (A) The relative POD-like activity of $FePO_4$@GO NPs catalytic system with ROS scavengers (IPA, BQ, D-Histidine and EDTA, 5 mM; FePO4@GO NPs: 0.10 mg/mL, TMB:1.0 mM, H_2O_2: 0.5 mM, pH 3.6. Inset: color changes of TMB in four samples).

Figure S2.

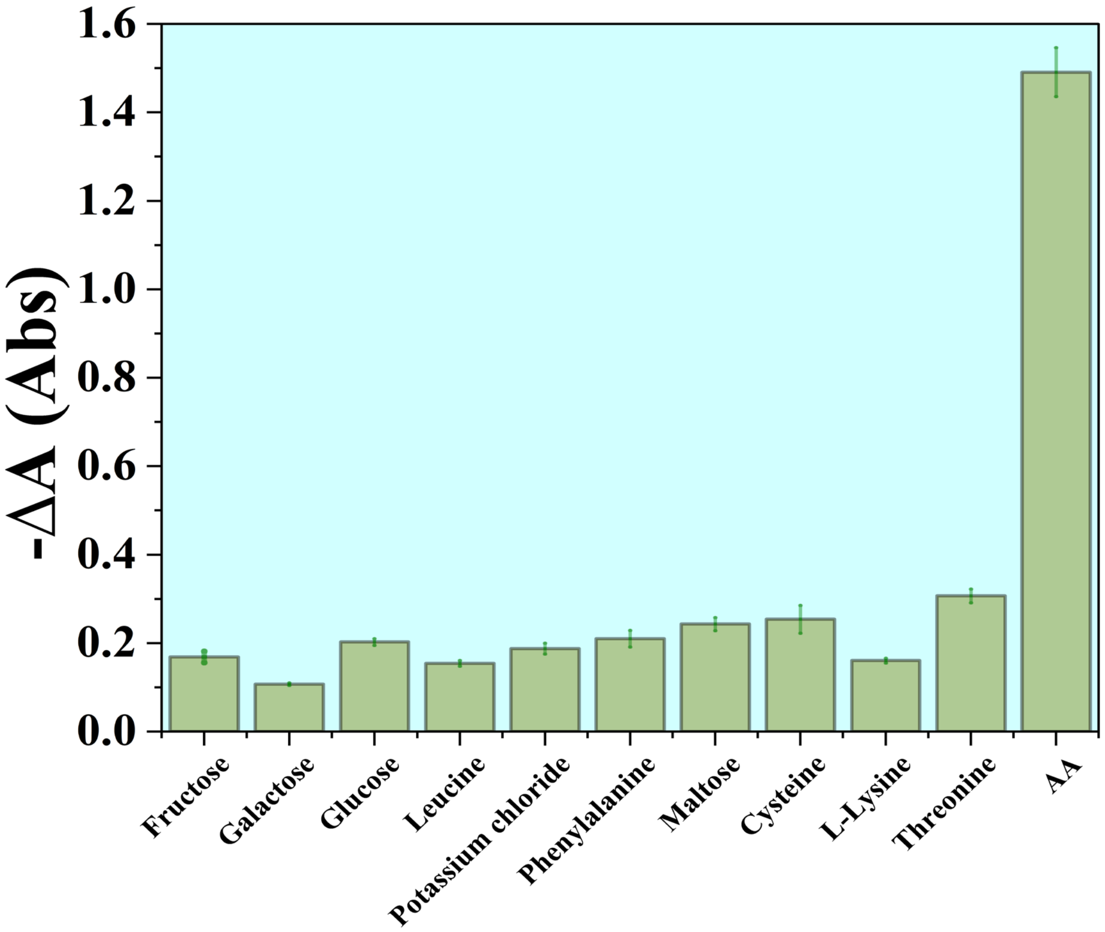

Figure S2. The selectivity of $FePO_4$@GO NPs for AA detection. (1 mM AA, 10 mM interfere materials).

Figure S3

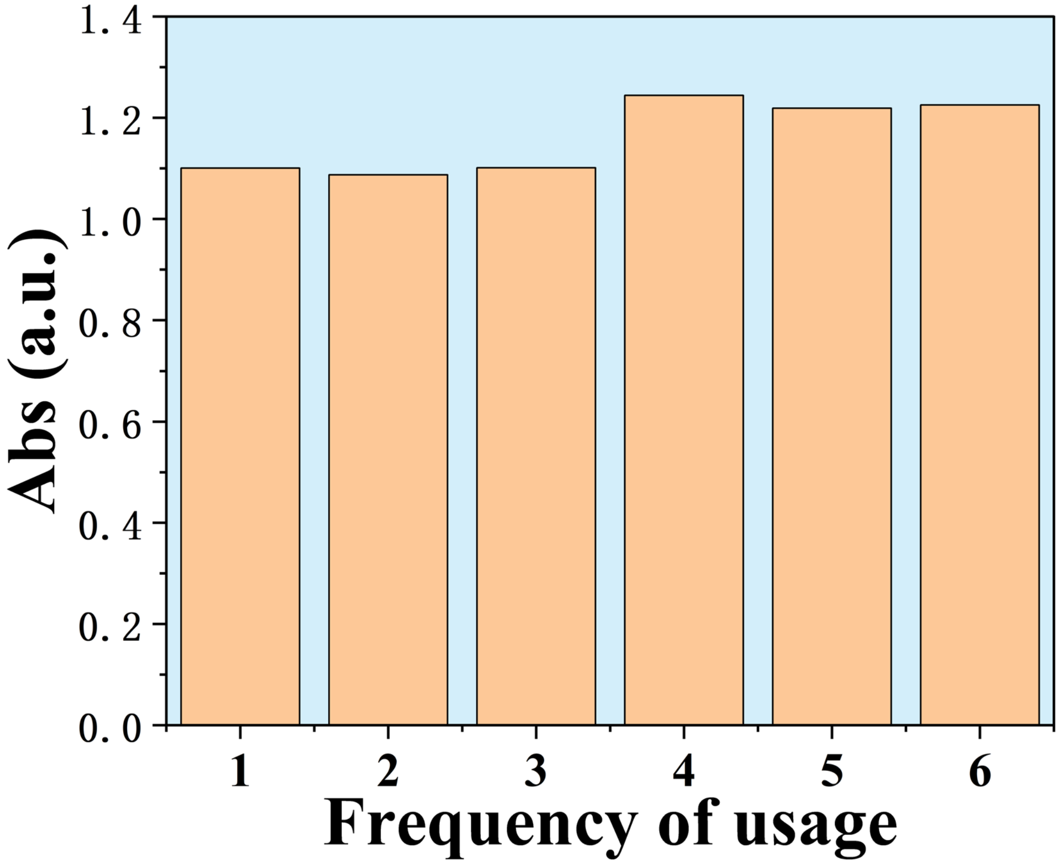

Figure S3. The UV-vis absorption spectra of recycling experiment on $FePO_4$@GO NPs.

Figure S4.

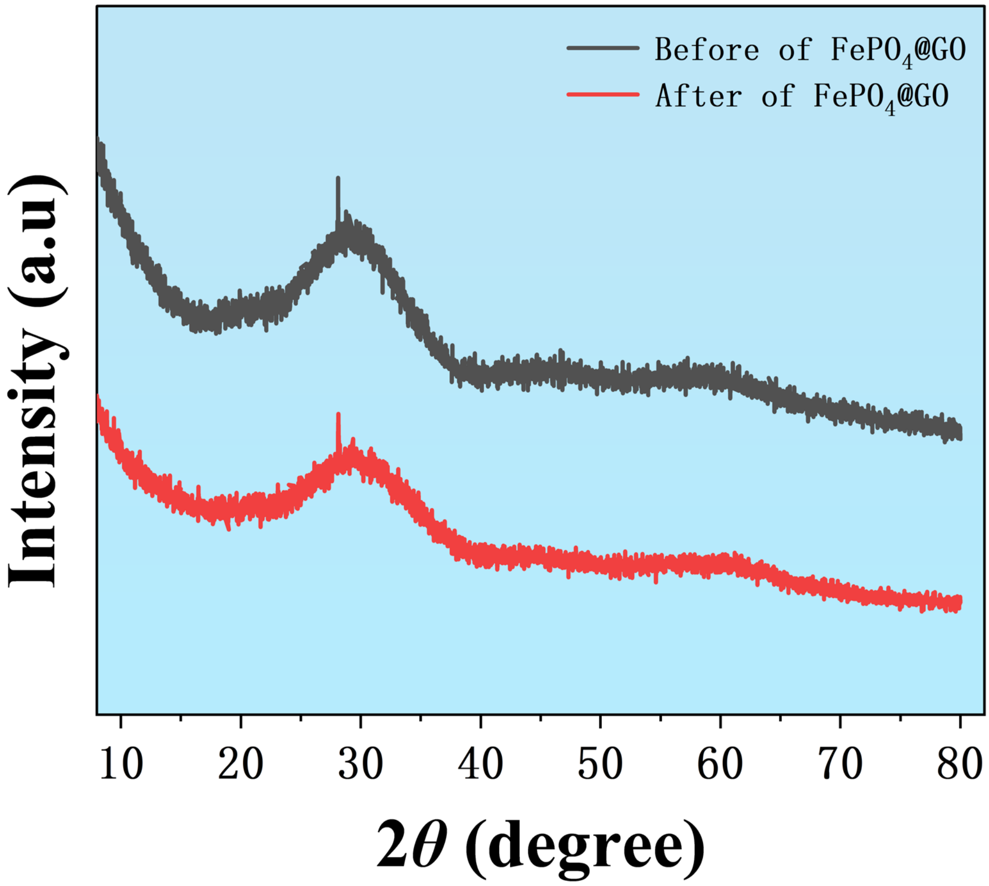

Figure S4. The XRD patterns of recycling experiment on $FePO_4$@GO NPs.

Table S1

**Enzyme Catalyst**	**K_m (mM)**	**v_{max} (10^{-8} M s^{-1})**	References
HRP	0.434	10	[1]
Pt NPs	0.48	79	[2]
MIL-53(Fe)	1.08	8.78	[3]
Fe3O4	0.098	3.44	[1]
h-Fe_3O_4@ppy	0.27	6.8	[4]
TpBTD	0.10	18.38	[5]
$FePO_4$@GO	0.2202	7.71219	This work

Table S1. Comparison of the Michaelis-Menten constant (K_m) and the maximum velocity (V_{max}) of FePO4@GO NPs with other nanomaterials.

Table S2

**Materials**	**Method**	**Linear range (mM)**	**LODs (μM)**	**Ref.**
Co_3O_4@β-CD NPs	Colorimetric	0.01-0.60	1.09	[6]
Co_3O_4 NPs	Colorimetric	0.01-0.35	3.91	[6]
Cu NPs@C	Colorimetric	0.01-1.00	141	[7]
Ni–Pt alloys	Electrochemical	0.57–5.68	570	[8]
CDs	Fluorescence	136-227	-	[9]
$FePO_4$@GO	Colorimetric	2.5-75	1.25	This work

Table S2. Comparison of AA detection with different methods.

References

1. Gao, L.; Zhuang, J.; Nie, L.; Zhang, J.; Zhang, Y.; Gu, N.; Wang, T.; Feng, J.; Yang, D.; Perrett, S.; Yan, X., Intrinsic peroxidase-like activity of ferromagnetic nanoparticles. *Nature Nanotechnology* **2007,** 2, (9), 577-583.
2. Xi, Z.; Wei, K.; Wang, Q.; Kim, M. J.; Sun, S.; Fung, V.; Xia, X., Nickel–Platinum Nanoparticles as Peroxidase Mimics with a Record High Catalytic Efficiency. *Journal of the American Chemical Society* **2021,** 143, (7), 2660-2664.
3. Ai, L.; Li, L.; Zhang, C.; Fu, J.; Jiang, J., MIL-53(Fe): A Metal–Organic Framework with Intrinsic Peroxidase-Like Catalytic Activity for Colorimetric Biosensing. *Chemistry – A European Journal* **2013,** 19, (45), 15105-15108.
4. Jampaiah, D.; Srinivasa Reddy, T.; Kandjani, A. E.; Selvakannan, P. R.; Sabri, Y. M.; Coyle, V. E.; Shukla, R.; Bhargava, S. K., Fe-doped CeO2 nanorods for enhanced peroxidase-like activity and their application towards glucose detection. *Journal of Materials Chemistry B* **2016,** 4, (22), 3874-3885.
5. Liang, L.; Jiang, Y.; Liu, F.; Wu, J.; Tian, L.; Zhao, S.; Ye, F., Smartphone flashlight-triggered covalent organic framework nanozyme activity: A universal scheme for visual point-of-care testing. *Sensors and Actuators B: Chemical* **2023,** 381, 133422.
6. Lu, W.; Zhang, J.; Li, N.; You, Z.; Feng, Z.; Natarajan, V.; Chen, J.; Zhan, J., Co3O4@β-cyclodextrin with synergistic peroxidase-mimicking performance as a signal magnification approach for colorimetric determination of ascorbic acid. *Sensors and Actuators B: Chemical* **2020,** 303, 127106.
7. Tan, H.; Ma, C.; Gao, L.; Li, Q.; Song, Y.; Xu, F.; Wang, T.; Wang, L., Metal–Organic Framework-Derived Copper Nanoparticle@Carbon Nanocomposites as Peroxidase Mimics for Colorimetric Sensing of Ascorbic Acid. *Chemistry – A European Journal* **2014,** 20, (49), 16377-16383.
8. Weng, Y.-C.; Lee, Y.-G.; Hsiao, Y.-L.; Lin, C.-Y., A highly sensitive ascorbic acid sensor using a Ni–Pt electrode. *Electrochimica Acta* **2011,** 56, (27), 9937-9945.
9. Fong, J. F. Y.; Chin, S. F.; Ng, S. M., A unique “turn-on” fluorescence signalling strategy for highly specific detection of ascorbic acid using carbon dots as sensing probe. *Biosensors and Bioelectronics* **2016,** 85, 844-852.
